# Requirement of novel amino acid fragments of orphan nuclear receptor TR3/Nur77 for its functions in angiogenesis

**DOI:** 10.18632/oncotarget.4637

**Published:** 2015-06-25

**Authors:** Yan Li, Pierre M. Bourbon, Marianne A. Grant, Jin Peng, Taiyang Ye, Dezheng Zhao, Huiyan Zeng

**Affiliations:** ^1^ Center for Vascular Biology Research and Division of Molecular and Vascular Medicine, Department of Medicine, Beth Israel Deaconess Medical Center and Harvard Medical School, Boston, MA, USA; ^2^ Department of Gastroenterology, Shandong Provincial Hospital Affiliated to Shandong University, Ji-nan, PR China; ^3^ Department of Pathology, Beth Israel Deaconess Medical Center and Harvard Medical School, Boston, MA, USA; ^4^ Department of Medical Oncology and Radiation Oncology, Zhongnan Hospital of Wuhan University, Wuhan, PR China; ^5^ Department of Obstetrics and Gynecology, Renji Hospital, Shanghai Jiao Tong University, School of Medicine, Shanghai, PR China; ^6^ Division of Gastroenterology, Department of Medicine, Beth Israel Deaconess Medical Center and Harvard Medical School, Boston, MA, USA

**Keywords:** actin stress fibers, migration, permeability, proliferation, protein binding site

## Abstract

Pathological angiogenesis is a hallmark of many diseases. We demonstrated that TR3/Nur77 is an excellent target for pro-angiogenesis and anti-angiogenesis therapies. Here, we report that TR3 transcriptionally regulates endothelial cell migration, permeability and the formation of actin stress fibers that is independent of RhoA GTPase. 1) Amino acid residues 344-GRR-346 and de-phosphorylation of amino acid residue serine 351 in the DNA binding domain, and 2) phosphorylation of amino acid residues in the 41-61 amino acid fragment of the transactivation domain, of TR3 are required for its induction of the formation of actin stress fibers, cell proliferation, migration and permeability. The 41-61 amino acid fragment contains one of the three potential protein interaction motifs in the transactivation domain of TR3, predicted by computational modeling and analysis. These studies further our understanding of the molecular mechanism, by which TR3 regulates angiogenesis, identify novel therapeutic targeted sites of TR3, and set the foundation for the development of high-throughput screening assays to identify compounds targeting TR3/Nur77 for pro-angiogenesis and anti-angiogenesis therapies.

## INTRODUCTION

Pathological angiogenesis is a hallmark of many diseases including cancer, inflammation, wound healing and ischemic heart disease. Anti-VEGF neutralizing antibodies and VEGFR kinase/multiple kinase inhibitors have been successfully developed and widely used in the clinic (reviewed in [1]). However, in addition to their toxic side effects [2], VEGF-targeted therapies in cancer face the problems of insufficient efficacy [3-12], resistance and intrinsic refractoriness [10, 13, 14]. Therefore, it is desirable to identify additional angiogenesis targets. Our recent studies demonstrated that TR3/Nur77 (human: TR3, mouse: Nur77, rat: NGFI-B) is one such promising target [15-17].

TR3/Nur77 is a member of nuclear receptor IV subfamily of transcription factors, without physiological ligand [18], although several agonists, cytosporone B and a series of methylene-substituted diindolymethanes, were identified [19, 20]. The nuclear receptor IV subfamily members play redundant roles in TCR-mediated apoptosis [21] and brown fat thermogenesis [22, 23]. However, they play different roles in development (reviewed in [24]). TR3/Nur77 also plays important roles in cancer cell biology, inflammation, metabolism diseases, stress and addiction (reviewed in [25-28]).

Prior to our recent studies [16], little was known about the role of TR3/Nur77 in angiogenesis. Our studies demonstrated that TR3/Nur77 is a critical mediator of angiogenesis. We found that TR3/Nur77 is highly and transiently up-regulated in cultured endothelial cells (EC) and during angiogenesis *in vivo.* TR3 is induced by angiogenic factors having microvessel permeable activity, including VEGF, histamine and serotonin, but not by angiogenic factors that do not have microvessel permeable activity, including bFGF, PlGF and PDGF [15-17], and in postnatal angiogenesis, such as tumor angiogenesis and skin wound healing [16, 29]. *In gain of function assays*, overexpression of TR3/Nur77 cDNA is sufficient to induce endothelial cell proliferation, migration and tube formation *in vitro.* Angiogenesis, microvessel permeability and normal skin wound healing are greatly induced/improved in our transgenic EC-Nur77-S mice, in which the full-length Nur77 cDNA is inducibly and specifically expressed in mouse endothelium [15-17]. The transgenic EC-Nur77-S mice are healthy after Nur77 has been induced for three months [29]. *In loss of function assays*, knockdown of TR3 expression by its antisense DNA or shRNA inhibits endothelial cell proliferation, migration and tube formation induced by VEGF, histamine and serotonin *in vitro*. Tumor growth, angiogenesis and microvessel permeability induced by VEGF, histamine or serotonin are almost completely inhibited in Nur77 knockout mice [15-17]. Paradoxically, however, Nur77 null mice are viable, fertile, appear to develop a normal adult vasculature and have no defect in normal skin wound healing [21, 29]. Our studies demonstrated that TR3/Nur77 is an excellent target for pro-angiogenesis and anti-angiogenesis therapies.

Our studies further demonstrated that TR3/Nur77 regulates angiogenesis in the early stage [15-17]. In adult vessels, vascular integrity is maintained by endothelial cell-endothelial cell (EC-EC) junctions and endothelial cell-basement membrane (EC-BM) interactions that are regulated by integrins. In order to induce angiogenesis, both of these interactions must be altered to facilitate endothelial cell proliferation and migration. Further, actin cytoskeleton regulates EC-EC junctions through its links to EC junctions and forms focal adhesion with integrins [30]. Recently, we reported that TR3 regulates the expression of eNOS, protein components in VE-cadherin associated adherent junctions and integrin β4 to induce angiogenesis [17, 29]. However, it is still not known whether TR3/Nur77 regulates the actin cytoskeleton.

It is known that TR3 plays different roles depending on its cellular localization (reviewed in [31]). When present in the nucleus, TR3 functions as a transcription factor that regulates gene expression and promotes cell growth. In the cytosol, TR3 does not have transcriptional activity, but associates with other proteins, such as PKC to inhibit PKC activity [32] or Bcl2 to promote cell apoptosis [31]. Previously, we reported that both of the transcriptional activation domain and the DNA-binding domain, but not the ligand-binding domain, of TR3 are required for its regulation of angiogenesis and gene expression [16, 17, 29]. However, the structure and functional relationship of TR3 in angiogenesis is still not completely investigated. In this study, we find that TR3 regulates the formation of actin stress fibers and this effect does not involve RhoA GTPase. Study of the structure and functional relationship indicates that 1) amino acid residues 344-GRR-346 and de-phosphorylation of amino acid residue serine 351 in the DNA binding domain, and 2) phosphorylation of the amino acid residues within the amino acid fragment 41-61 in the transactivation domain are required for TR3-regulated the formation of actin stress fibers, cell proliferation, migration and permeability.

## RESULTS

### TR3 regulates endothelial cell morphology and the formation of actin stress fibers, independent of RhoA

**Figure 1:**
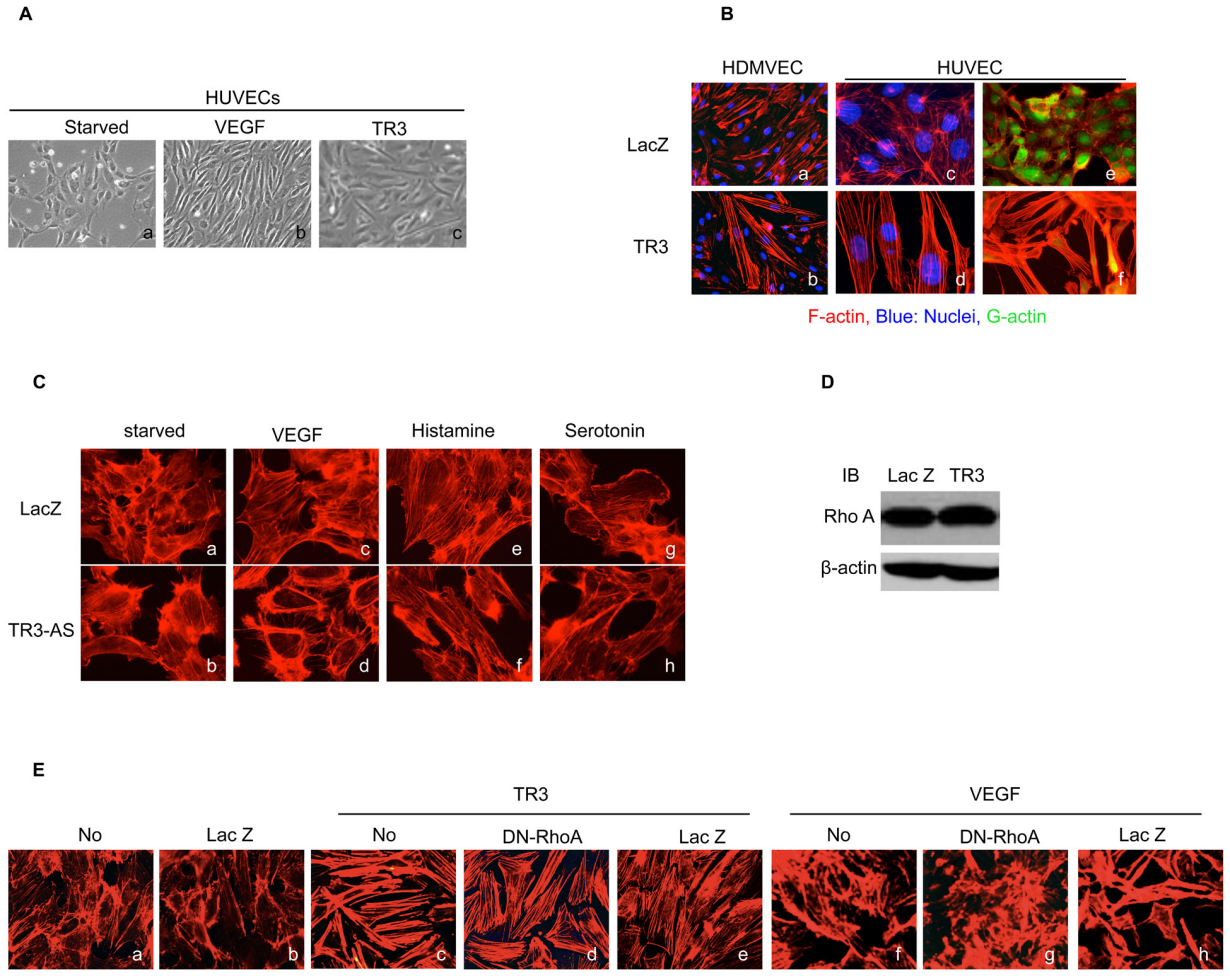
TR3 regulates endothelial cell morphology and the formation of actin stress fibers, independent of RhoA **A.** Serum-starved HUVECs were treated with (b) or without (a) VEGF (50 ng/ml), or infected with virus expressing TR3 (c) for three days and photographed; **B.** HDMVECs (a and b) or HUVECs (c, d, e and f) were transduced with Lac Z as control (a, c and e) or TR3 (b, d and f), and stained with rhodamine phalloidin without (a, b, c and d) or with DNase I (e and f); **C.** HUVECs that were transduced with viruses expressing Lac Z as control or TR3 antisense DNA (TR3-AS) were serum starved for 24 hours, stimulated without (a and b), or with 10 ng/ml VEGF (c and d), 10 μM histamine (e and f) or 10 μM serotonin (g and h) for 30 minutes and stained with rhodamine phalloidin. **D.** Cellular extracts from HUVECs that were transduced with viruses expressing Lac Z as control or TR3 were subjected to immunoblotting with antibodies against RhoA (top panel) or β-actin as control for protein equal loading (bottom panel); **E.** HUVECs without transduction (a and f), transduction with Lac Z (b and h), TR3 (c), TR3 + RhoA dominant negative mutant (DN-RhoA) (d), TR3 + Lac Z as control (e), DN-RhoA (g), treated without (a-e) and with 10 ng/ml VEGF (f-h) were stained with rhodamine phalloidin. Experiments were repeated three times.

Although the effects of TR3 on endothelial cell proliferation and migration have been studied previously [15-17], it is not known how TR3 affects endothelial cell morphology and what the structure and function relationship lies behind this effect. In the present study, we find that, compared to the control Lac Z-expressing Human Umbilical Vein Endothelial Cells (HUVEC), cells infected with TR3-expressing viruses are elongated, with some similarity to VEGF-stimulated cells (Figure 1A). It is known that cell morphology is regulated by three types of actin cytoskeleton structures, including filopodia, lamellipodia and stress fibers. Filopodia and lamellipodia are required for stabilization of endothelial cell adherent junctions, whereas formation of stress fibers is associated with disruption of endothelial integrity [33-35]. As VEGF induces the formation of actin stress fibers [36] and also stimulates TR3 expression, we wondered whether TR3 overexpression alone can mediate the formation of stress fibers. HUVECs and Human Dermal Microvascular Endothelial Cells (HDMVEC) were transduced with viruses expressing Lac Z as control, or TR3 cDNA (TR3). Two days later, cells were serum-starved for 24 hours and fixed with 4% paraformaldehyde and then stained with rhodamine phalloidin that specifically binds to filamentous actin (F-actin). The formation of filopodia, lamellipodia and stress fibers was examined by fluorescence microscopy. Filopodia and lamellipodia are filamentous membrane projections that contain long parallel actin filaments arranged in tight bundles and cytoplasmic protrusions that contain a thick cortical network of actin filaments, respectively, while stress fibers are bundles of actin filaments. Our results show that expression of TR3 induces the formation of long parallel actin filaments in both HUVECs and HDMVECs (Figure 1B, a *vs.* b, c *vs.* d). Then, we examined whether TR3 regulates globular actin (G-actin) by detection with fluorescent Deoxyribonuclease I (DNase I). G-actin is detected in all cells transduced with Lac Z, but almost undetectable in cells transduced with TR3 (Figure 1B, e and f). Next, we study whether expression of TR3 is required for the formation of actin stress fibers by using viruses expressing TR3 antisense DNA that can completely inhibit the endogenous TR3 expression [16]. HUVECs were transduced with Lac Z as control or TR3 antisense RNA (TR3-AS). Two days later, cells were serum-starved for 24 hours, stimulated with or without VEGF for 30 minutes, and then stained with rhodamine phalloidin. The formation of actin stress fibers induced by VEGF is completely inhibited by TR3 antisense RNA (Figure 1C, c *vs.* d). Recently, we reported that expression of TR3 is required for the angiogenesis and microvessel permeability induced by histamine and serotonin [15]. We further examined whether expression of TR3 is required for the induction of actin stress fibers induced by histamine and serotonin. Indeed, actin stress fibers induced by histamine and serotonin are almost completely inhibited in HUVECs transduced with TR3 antisense RNA (Figure 1C, e *vs.* f; g *vs.* h).

Since RhoA GTPase regulates the formation of actin stress fibers, we wondered whether TR3 regulates the expression of RhoA because TR3 is a transcription factor. HUVECs were transduced with viruses expressing Lac Z as control and TR3 cDNA. Cellular extracts were subjected to immunoblotting analysis with an antibody against RhoA. We find that TR3 does not regulate the expression of RhoA (Figure 1D). We further examined whether RhoA activity is required for the formation of actin stress fibers induced by TR3. HUVECs were transduced with or without viruses expressing Lac Z as control, TR3 cDNA, TR3 + RhoA dominant negative mutant (DN-RhoA) [37], or TR3 + Lac Z as control for RhoA, and then stained with rhodamine phalloidin. As shown in Figure 1E, expression of TR3 induces the formation of actin stress fiber (Figure 1E, c vs. b). To our surprise, DN-RhoA is unable to inhibit the formation of actin stress fibers induced by TR3 (Figure 1E, d *vs.* c). In order to confirm that the DN-RhoA is functional, HUVECs were transduced with or without viruses expressing DN-RhoA, or Lac Z as control, and then stimulated with VEGF. In the absence of any virus transduction, VEGF induces the formation of actin stress fiber (Figure 1E, f *vs.* a). The formation of actin stress fibers induced by VEGF is completely inhibited by DN-RhoA expression, but is not affected by the Lac Z as control (Figure 1E, g *vs.* f and h *vs.* f). Our results demonstrate that overexpression of TR3 protein induces the formation of actin stress fibers, while inhibition of TR3 expression by its antisense RNA prevents the formation of actin stress fibers. RhoA is not involved in the formation of actin stress fibers induced by TR3.

### Requirement of TR3 transcriptional activity for its regulation of actin stress fibers, cell migration and permeability

**Figure 2:**
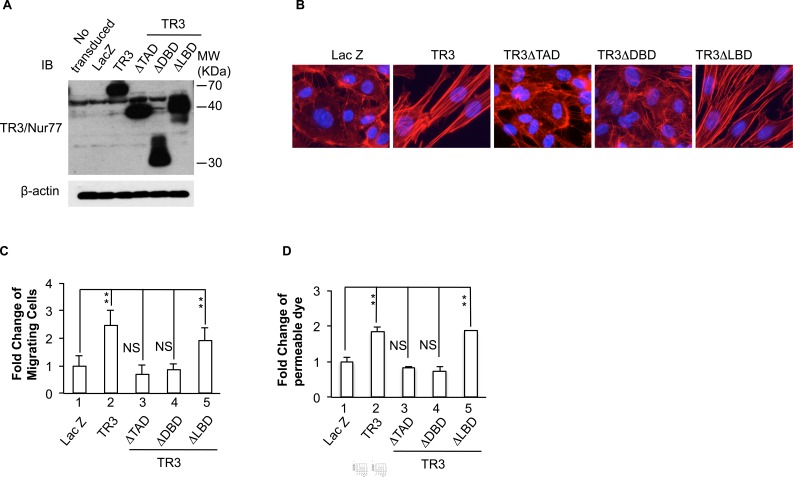
Requirement of the transcriptional activity of TR3 for its regulation of actin stress fibers, cell migration and permeability HUVECs that were transduced without or with Lac Z as control, TR3 full-length cDNA, TR3ΔTAD, TR3ΔDBD or TR3ΔLBD were subjected to immunoblotting with antibodies against TR3 (top panel) or β-actin as control for protein equal loading (bottom panel) **A.** staining with rhodamine phalloidin **B.** migration assay **C.** and permeability assay **D.** (**p < 0.005, NS, *p* > 0.05). Experiments were repeated three times.

TR3/Nur77 is an orphan member of the steroid/thyroid/retinoid superfamily whose members act mainly as transcription factors that induce or repress gene expression [38]. However, several groups [39-41] have recently shown that TR3's ability to induce apoptosis in tumor cells is independent of transcription and occurs when TR3 transmigrates from the nucleus to the cytoplasm. Like other members of its superfamily, TR3/Nur77 has three major domains that regulate different functions (Supplemental Figure 1S). These include the N-terminal transactivation domain and the DNA binding domain that are essential for regulating gene transcription, and a C-terminal ligand-binding domain. Following the work of Kolluri *et al.* [41], we prepared mutant forms of TR3, TR3ΔTAD, TR3ΔDBD and TR3ΔLBD that lacks each of these domains [16] (Supplemental Figure 1S). HUVECs were transduced with viruses expressing Lac Z as control, TR3 full-length cDNA, TR3ΔTAD, TR3ΔDBD or TR3ΔLBD. Cellular extracts were subjected to immunoblotting analysis. As shown in Figure 2, TR3, TR3ΔTAD, TR3ΔDBD and TR3ΔLBD are expressed at comparable levels (Figure 2A). HUVECs transduced with these viruses were subjected to rhodamine phalloidin staining. The results show that TR3ΔTAD and TR3ΔDBD are unable to induce the formation of actin stress fibers, but TR3ΔLBD induces the formation of actin stress fibers, similar to those induced by full-length TR3 (Figure 2B).

**Figure 3:**
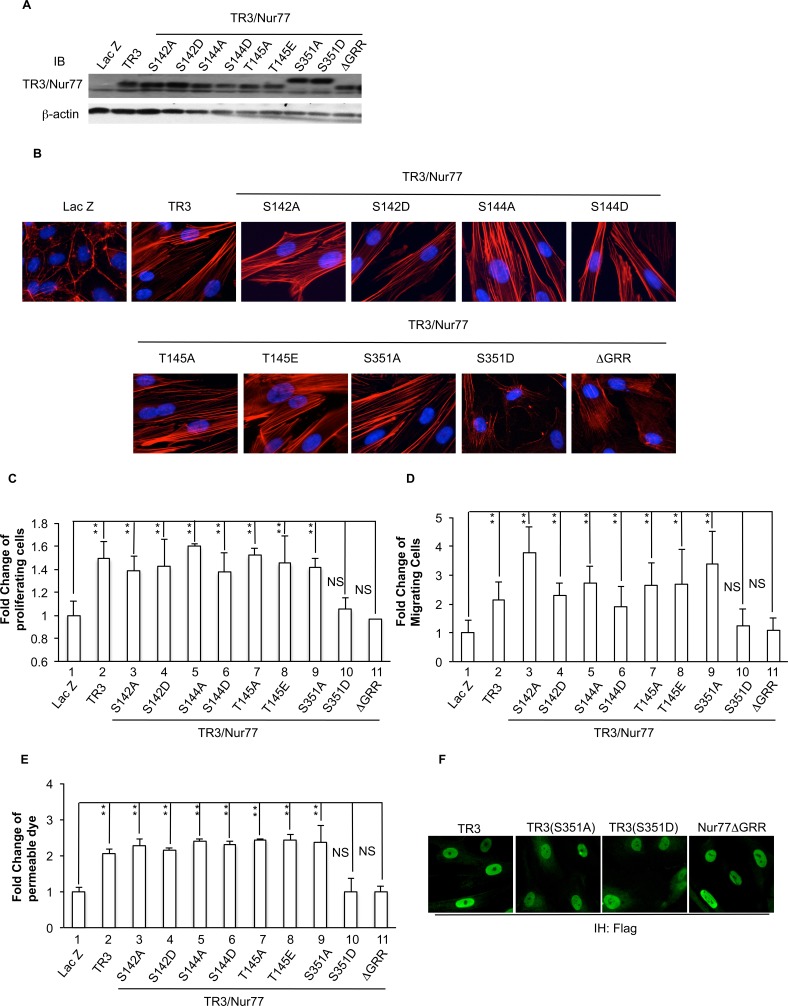
Requirement of Serine 351 de-phosphorylation and amino acid residues GRR in the DNA binding domain of TR3/Nur77 to induce cell proliferation, migration, monolayer permeability and the formation of actin stress fibers HUVECs that were transduced without or with Lac Z as control, TR3, Nur77(S142A), Nur77(S142D), Nur77(S144A), Nur77(S144D), Nur77(T145A), Nur77(T145E), TR3(S351A) and TR3(S351D) or Nur77ΔGRR were subjected to immunoblotting with antibodies against TR3/Nur77 (top panel) or β-actin as control for protein equal loading (bottom panel) **A.** staining with rhodamine phalloidin **B.** proliferation assay **C.** migration assay **D.** and permeability assay **E.** (**p < 0.005, NS, *p* > 0.05). **F.** Immunostaining of HUVEC expressing TR3, TR3(S351A) and TR3(S351D) or Nur77ΔGRR with an antibody against Flag. Experiments were repeated three times.

**Figure 4:**
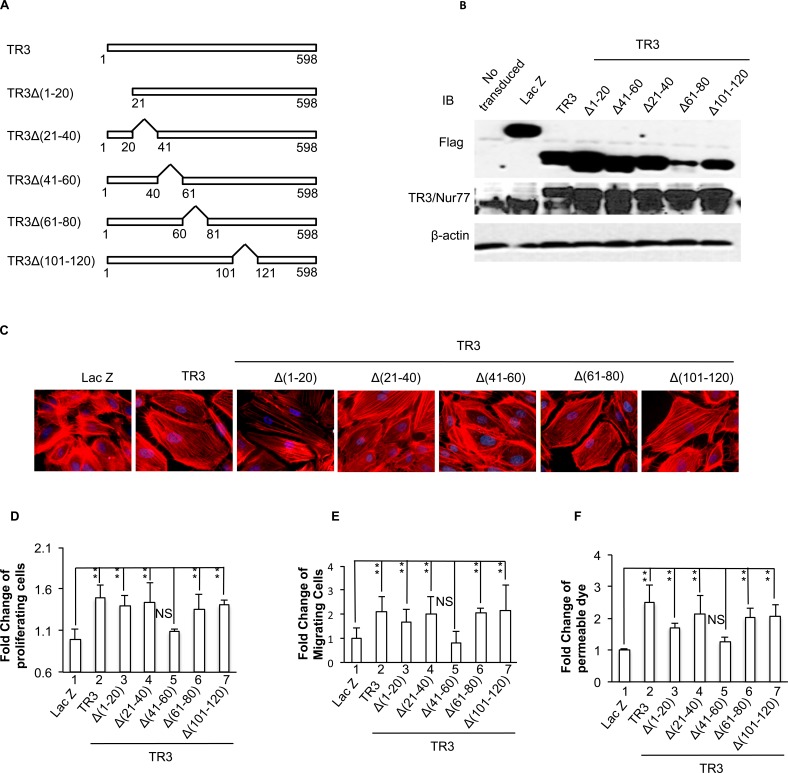
Requirement of amino acid 41-60 segment of TR3 for its regulation of cell proliferation, migration, monolayer permeability and the formation of actin stress fibers **A.** Diagram of TR3 deletion mutants; **B.**, **C.**, **D.**, **E.**, and **F.** HUVECs that were transduced without or with Lac Z as control, TR3, TR3Δ(1-20), TR3Δ(21-40), TR3Δ(41-60), TR3Δ(61-80) and TR3Δ(101-120) were subjected to immunoblotting with antibodies against Flag-tag (top panel), TR3 (middle panel) or β-actin as control for protein equal loading (bottom panel) **B.** staining with rhodamine phalloidin **C.** proliferation assay **D.** migration assay **E.** and permeability assay **F.** (**p < 0.005, NS, *p* > 0.05). Experiments were repeated three times.

**Figure 5:**
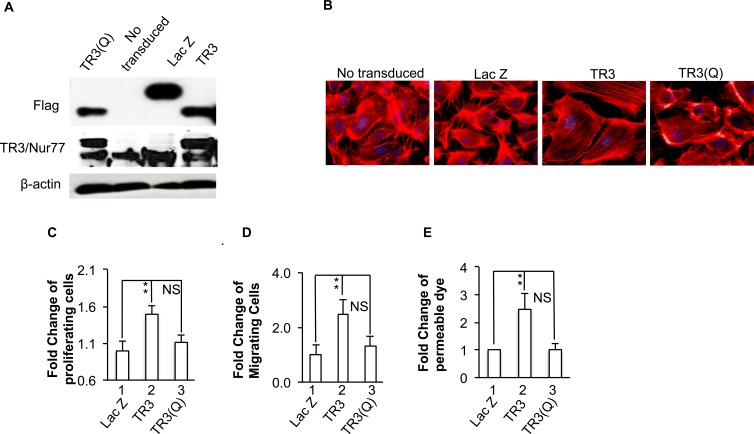
Phosphorylation of amino acid residues in 41-61 fragment is required for TR3-induced cell proliferation, migration, monolayer permeability and the formation of actin stress fibers HUVECs that were transduced without or with Lac Z as control, TR3, or TR3(Q) were subjected to immunoblotting with antibodies against Flag-tag (top panel), TR3 (middle panel) or β-actin as control for protein equal loading (bottom panel) **A.** staining with rhodamine phalloidin **B.** proliferation assay **C.** migration assay **D.** and permeability assay **E.** (**p < 0.005, NS, *p* > 0.05). Experiments were repeated three times.

Our previous studies indicated that TR3ΔTAD and TR3ΔDBD, but not TR3ΔLBD, are unable to induce cell proliferation, down-regulation of the proteins in the VE-cadherin adherence junctions, and up-regulation of e-NOS and integrin β4 [17, 29]. However, it is not known whether the transcriptional activity of TR3 is required for its regulation of cell migration and permeability. HUVECs were transduced with viruses expressing Lac Z as control, TR3 full-length cDNA, TR3ΔTAD, TR3ΔDBD or TR3LBD and subjected to scratch migration assay and monolayer permeability assay. In both assays, TR3ΔTAD and TR3ΔDBD are unable to induce migration or permeability (Figure 2C and 2D, 3 and 4 *vs.* 1, *p* > 0.05 and Supplemental Figure 2S). TR3ΔLBD induces migration and permeability about 2 fold as comparing to Lac Z as control, in a similar way as TR3 does (Figure 2C and 2D, 5 and 2 *vs.* 1, *p* < 0.005 and Supplemental Figure 2S). These data demonstrate that the transcriptional activation domain and the DNA binding domain, but not the ligand-binding domain of TR3, are required for its induction of cell migration, permeability and the formation of actin stress fibers.

### De-phosphorylation of serine 351 and amino acid residues GRR in the DNA binding domain of TR3 are required for its induction of cell proliferation, migration, monolayer permeability and the formation of actin stress fibers

Our previous and above studies demonstrated that the TR3ΔTAD mutant and the TR3ΔDBD mutant, in which amino acids 1-167 (containing transactivation domain) or 168-367 (containing DNA binding domain) were deleted, lost the ability to regulate the expression of its targeted genes, the formation of actin stress fibers, cell proliferation, migration, monolayer permeability and angiogenesis ([15-17] and Figure 2). Others reported that the fragment (74-174 amino acid residues) within the AF-1 domain of Nur77 is required for NurRE binding [42-44]. Mutation of the serine and threonine residues within the 74-174 amino acid residues indicated that serine 142 residue of Nur77 is required for neuron differentiation [44]. The threonine 145 residue of Nur77 is phosphorylated by Erk, but its function is not known [44]. The function of the serine 144 residue of Nur77 has not been studied. The amino acid residue serine 351 within the TR3 DNA binding domain was shown to be able to be phosphorylated by Akt, but its function in angiogenesis is not known [41, 45]. We generated mutants of Nur77(S142A), Nur77(S142D), Nur77(S144A), Nur77(S144D), Nur77(T145A), Nur77(T145E), TR3(S351A) and TR3(S351D), in which serine 142, serine 144, threonine 145 in mouse Nur77, or serine 351 in human TR3 are mutated to alanine, aspartic acid or glutamic acid, to prevent phosphorylation and mimic the function of phosphorylation, respectively. These mutants were expressed at similar levels in HUVECs with our viral expression system (Figure 3A) and subjected to analysis of actin stress fibers, cell proliferation, migration, and monolayer permeability assays. The data show that similar to TR3, all of the Nur77(S142A), Nur77(S142D), Nur77(S144A), Nur77(S144D), Nur77(T145A) and Nur77(T145E) mutants in the transactivation domain retain the ability to induce morphological change (Supplemental Figure 3S), the formation of actin stress fibers (Figure 3B), cell proliferation, migration and permeability, as TR3 does (Figure 3C, 3D and 3E, 2-8 *vs.* 1, *p* <0.005 and Supplemental Figure 4S). The TR3(S351A) mutation is not defective in the formation of actin stress fiber (Figure 3B), cell proliferation, migration and permeability (Figure 3C, 3D and 3E, 9 *vs.* 1, *p* < 0.005; and Supplemental Figure 4S). However, TR3(S351D) and the deletion mutant Nur77ΔGRR, in which three amino acid residues GRR (347-349) in the DNA binding domain of Nur77 are deleted (equivalent to GRR(344-346) in human TR3), are unable to induce the formation of actin stress fibers (Figure 3B), cell proliferation, migration and permeability (Figure 3C, 3D and 3E, 10 and 11 *vs.* 1, p > 0.05, and Supplemental Figure 4S). Our results demonstrate that GRR(344-346) residues and de-phosphorylated S351 in the DNA binding domain of TR3, are required for TR3-mediated formation of actin stress fibers, cell proliferation, migration and permeability, but none of the known phosphorylable residues in the AF-1 domain of TR3 are required for the formation of actin stress fibers, cell proliferation, migration and permeability induced by TR3.

It was well known that TR3/Nur77 has several different functions which appear to depend on its cellular localization (reviewed in [31]). We further study whether the lost function of TR3(S351D) and Nur77ΔGRR is due to their cellular localization. HUVEC were transduced with TR3 full-length cDNA, TR3(S351A) and TR3(S351D) or Nur77ΔGRR and then immunostained with an antibody against Flag. As shown in Figure 3F, all of proteins are detected in nuclei (Figure 3F). Our data demonstrate that inability to induce actin stress fibers, cell proliferation, migration and permeability of TR3(S351D) and Nur77ΔGRR is not due to alteration of their cellular localization.

### Requirement of the amino acid 41-60 segment of TR3 for its regulation of the formation of actin stress fibers, cell proliferation, migration and monolayer permeability

We then generated serial deletion mutants, TR3Δ(1-20), TR3Δ(21-40), TR3Δ(41-60), TR3Δ(61-80) and TR3Δ(101-120), in which amino acid residues 1-20, 21-40, 41-60, 61-80 and 101-120 of TR3 were deleted (Figure 4A). These mutants were expressed to similar levels in HUVECs (Figure 4B) and subjected to actin stress fiber analysis, cell proliferation, migration, and monolayer permeability assays. The data show that TR3Δ(41-60) is unable, but TR3Δ(1-20), TR3Δ(21-40), TR3Δ(61-80) and TR3Δ(101-120), in a manner similar to TR3, are able to induce the formation of actin stress fibers (Figure 4C), cell proliferation, migration and permeability (Figure 4D, 4E and 4F, 5 *vs.* 1, p > 0.05, all others *vs.* 1, *p* < 0.005, and Supplemental Figure 5S). These data indicate that the amino acid residues 41-60 is required for the angiogenic responses of TR3.

### Phosphorylation of Thr 48, 55 and 61, Ser 52 and 54, and Tyr 60 is required for TR3-induced cell proliferation, migration, monolayer permeability and the formation of actin stress fibers

Considering that phosphorylation is the most important form of covalent modification for TR3 subfamily members, we hypothesized that phosphorylation of several amino acid residues within the amino acid 41-60 region may regulate the angiogenic responses of TR3. As the fragment (aa41-61) (EAAPAAPTALPSFSTFM DGYT) of TR3 has six potential phosphorylation sites, we replaced all of the threonine residues and serine residues with alanine, and tyrosine with phenylalanine, to generate a mutant TR3(Q) containing T48A-S52A-S54A-T55A-Y60F-T61A, mimicking the de-phosphorylated status of TR3. HUVECs were transduced with or without viruses expressing Lac Z as control, TR3 and TR3(Q). Cellular extracts were immunoblotted with antibodies against Flag tag and TR3. As shown in Figure 5, Lac Z, TR3 and TR3(Q) are expressed at similar levels (Figure 5A). Then, HUVECs transduced with or without viruses expressing Lac Z as control, TR3 and TR3(Q) were subjected to actin stress fiber analysis, cell proliferation, migration, and monolayer permeability. As shown in Figure 5, TR3(Q) loses the ability to induce the formation of actin stress fibers (Figure 5B), cell proliferation, migration and permeability (Figure 5C, 5D and 5E, 2 *vs.* 1, *p* < 0.005; 3 *vs.* 1, p > 0.05), suggesting that phosphorylation of amino acid residues in the 41-61 fragment of TR3 is required for its regulation of angiogenic responses.

### Protein structure and binding site prediction for TR3 N-terminal transactivation domain

**Figure 6:**
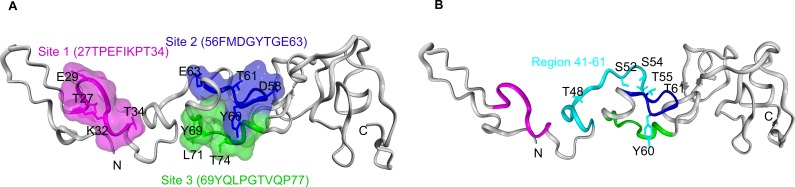
Protein structure and binding site prediction for TR3 N-terminal transactivation domain Sequence-based protein structure prediction of the N-terminal transactivation domain of human TR3, residues 1-167. **A.** Results from protein secondary structure, protein-binding regions in disordered proteins, and protein interaction interface residue predictions were complied and rendered on a representation of the predicted structure. Main chain backbone of residues of the N-terminal binding domain is shown in grey. Three sub-regions with ligand binding potential based on binding site and interaction interface residue predictions are shown with main chain rendering and space filled representations: site 1 (T27-T34) in magenta, site 2 (F56-E63) in blue, and site 3 (Y69-P77) in green. The side chains of residues identified computationally as having ligand binding potential are shown with labeled stick representations. **B.** The N-terminal transactivation domain of human TR3 as in (A) shown highlighting the region 41-60 in cyan, adjacent to and overlapping with site 2, in blue. Mutated residues Thr 48, 55 and 61, Ser 52 and 54, and Tyr 60, are shown with labeled stick representations in cyan.

Although the crystal structures of the DNA binding domain and the ligand-binding domain of TR3 have been determined [18, 46], there is no reported structure of its transactivation domain, nor structure of a homologous domain from a related protein family member. We studied whether our mutation results correlated with structure-function motifs in the transactivation domain of TR3 that are predicted by computational structure modeling and analysis. The sequence from human TR3, residues 1-167, was computationally assessed for protein secondary structure prediction with PSIPRED Protein Sequence Analysis [47], protein binding region prediction in disordered proteins (ANCHOR [48-50]), interface residue predictions (BSpred [51]) and protein structure prediction (GalaxyWEB [52]). The results from all predictions were correlated and rendered on a representation of the predicted structure of the N-terminal transactivation domain (Figure 6). The main chain backbone of residues of the N-terminal binding domain from 1-167 is shown in grey. Note, little secondary structure is predicted for this N-terminal sequence. Binding site and interaction interface residue predictions suggest that three sub-regions flanking the mutagenic region possess significant ligand binding potential: site 1 (T27-T34), site 2 (F56-E63), and site 3 (Y69-P77) (Figure 6A). These surface exposed and loop-containing regions are shown with main chain and space filled representations colored magenta (site1), blue (site 2), and green (site 3), respectively. The side chains of residues identified computationally as having significant ligand binding potential within each sub-region are shown with labeled stick representations. Sequence 41-60, deletion of which lost the activity to induce the actin stress fibers, proliferation, migration and permeability, contains site 2 (F56-E63). Site 1 (T27-T34) and site 3 (Y69-P77) are located in the 21-40 and 60-80 fragments of TR3, respectively, deletion of which has no effect on the formation of actin stress fibers, proliferation, migration and permeability. Residues Thr 48, 55 and 61, Ser 52 and 54, and Tyr 60, mutation of which to alanine lose the ability to induce the formation of actin stress fibers, proliferation, migration and permeability, are localized to a solvent-exposed loop comprising a sub-region of significant ligand-binding potential in the structural model, where residue side chains, with the exception of Ser 52, are potentially located on the protein surface (Figure 6B). Overall, the computational protein modeling and mutagenic analysis of the N-terminal binding domain of TR3 reveals the potential for interacting protein binding within a surface exposed and contiguous surface area within the domain predicted structure.

## DISCUSSION

The main findings of the present studies are 1) overexpression of TR3 induces the formation of actin stress fibers; 2) endogenous TR3 knockdown largely inhibits the formation of actin stress fibers induced by VEGF, histamine and serotonin; 3) RhoA does not play a role in the formation of actin stress fibers induced by TR3; 4) the transcriptional activity of TR3 is required for its induction of actin stress fibers; 5) study of its structure and function relationship indicates that the amino acid residues GRR(344-346) and the de-phosphorylation of serine 351 in the DNA binding domain of TR3, are required for the formation of actin stress fiber, cell proliferation and migration; 6) the amino acid fragment 41-60 within the transactivation domain of TR3 is required for the formation of actin stress fibers, cell proliferation, cell migration and permeability; 7) mutation of all potential phosphorylation sites in the fragment 41-61 of TR3 blocks its ability to induce the formation of actin stress fibers, cell proliferation, cell migration and permeability; and 8) computational modeling predicts that the transactivation domain of TR3 contains three potential protein interaction motifs located within the amino acid residues 27-34, 56-63 and 69-77 of TR3, respectively.

Our current studies demonstrate that overexpression of TR3/Nur77 induces the formation of actin stress fibers and TR3/Nur77 anti-sense DNA inhibits the formation of actin stress fibers induced by VEGF, histamine and serotonin (Figure 1). It is concerning that the formation of actin stress fibers occurs 30 minutes after stimulation, yet TR3 is up-regulated at 1 hour after stimulation by VEGF, histamine and serotonin [16, 17]. It is impossible that VEGF induces TR3 expression, resulting in the formation of actin stress fibers, because it takes 1 hour to detect the up-regulation of TR3 induced by VEGF, histamine and serotonin [16, 17]. It is possible that the expression of a protein(s) that is/are required for the formation of actin stress fibers induced by VEGF, histamine and serotonin does not exist in HUVECs that are transduced with TR3 antisense DNA, because TR3 is a transcription factor to regulate gene expression and the transcriptional activity of TR3 is required for its function in angiogenesis [15-17, 29]. We further found that the transcriptional activity of TR3 is indeed required for its induction of the formation of actin stress fibers (Figure 2), supporting the possibility that TR3 antisense DNA inhibits the expression of a protein(s) that is/are required for the formation of actin stress fibers induced by VEGF, histamine and serotonin. However, TR3 does not regulate the expression of RhoA, a key player in the formation of actin stress fibers. We further find that inhibition of RhoA activity with RhoA dominant negative mutant is unable to inhibit the formation of actin stress fibers induced by TR3, although the RhoA dominant negative mutant inhibits the formation of actin stress fibers induced by VEGF (Figure 1). These results suggest a novel mechanism, by which TR3 regulates the formation of actin stress fibers.

In adult vessels, vascular integrity is maintained by endothelial cell-endothelial cell (EC-EC) junctions and endothelial cell-basement membrane (EC-BM) interactions that are regulated by integrins (reviewed in [53]). Previously, we reported that TR3 regulates the expression of eNOS, protein components in the VE-cadherin associated adherence, including VE-cadherin, β-catenin, γ-catenin and p120, and integrin 4, to induce angiogenesis and microvessel permeability [17, 29]. Current studies demonstrate that TR3 induces the formation of actin stress fibers (Figure 1). These studies further our understanding of the molecular mechanism, by which TR3 regulates angiogenesis in early stage to induce endothelial cell permeability, proliferation and migration via destabilization of endothelial cell-endothelial cell (EC-EC) junctions and endothelial cell-basement membrane (EC-BM) interactions.

TR3/Nur77 has several different functions which appear to depend on its cellular localization (reviewed in [31]). When present in the nucleus, it acts as a transcription factor that regulates gene expression and promotes cell growth. In the cytosol, however, TR3/Nur77 is hyper-phosphorylated and does not have transcriptional activity, but associates with other proteins to promote cell apoptosis (reviewed in [31]). Flaig *et al.* reported that conformation of ligand binding domain is the structural basis for the cell-specific activities of NGFI-B [18]. Paulsen *et al.* has defined that a truncated mutant of Nur77 lacking amino acids 414-597 had similar transcriptional activities to wild-type Nur77 in a number of mammalian cell lines [54]. We reported that deletions of the transactivation domain and the DNA binding domain, TR3ΔTAD and TR3ΔDBD, but not the ligand-binding domain of TR3, TR3ΔLBD, lose theirregulation of endothelial cell proliferation and expression of EC-EC junction proteins, eNOS and integrin β4 [16, 17, 29]. Here, we show that these mutants play similar roles in cell migration and the formation of actin stress fibers and permeability (Figure 2). Further, TR3ΔTAD and TR3ΔLBD localize in nuclei like TR3 full-length cDNA [16]. Therefore, our studies demonstrate that the lost function of TR3 mutants are not due to their different cellular localization and further confirm that TR3/Nur77 regulates angiogenesis by its transcriptional activity.

Analysis of the crystal structure of the TR3 DNA binding domain indicates that an A box (344-GRRGRLPS-351 of TR3) in the C-terminal extension of NGFI-B/TR3 is important for monomeric binding to DNA by interacting with the DNA's minor groove [46]. It was reported that 1) phosphorylation of the Nur77 DNA binding domain mainly happens on serine residues; 2) phosphorylation of TR3 Ser 351 is stronger than that of Ser 341; and 3) phosphorylation of Ser 351 results in decrease of the affinity of TR3 for the NGFI-B response element (NBRE) while phosphorylation of Ser 341 does not [42]. Our results that TR3(S351A) enhances, whereas TR3(S351D) and Nur77ΔGRR cannot induce, HUVEC proliferation, migration and the formation of actin stress fibers, correlate very well with the crystal structure in such a way that the negative acidic group in the TR3(S351D) mutant prevents, and the deletion of positive arginine residues in Nur77ΔGRR loses, DNA interaction. These results further confirm that the DNA binding is required for TR3-regulated angiogenesis.

The TR3 mutant lacking the transactivation domain is unable to induce proliferation, migration, and the formation of actin stress fibers in HUVECs ([16] and Figure 2). Ser142 of TR3 is required for neuron differentiation and threonine 145 of Nur77 is phosphorylated by Erk [44]. However, our data indicate that these two amino acid residues and serine residue 144 do not play a role in TR3-regulated angiogenic responses (Figure 3). Instead, our studies identify a novel amino acid fragment 41-60 that is required for TR3-mediated angiogenic responses, because deletion of this fragment loses the ability to induce the formation of actin stress fibers, cell proliferation, migration, and permeability (Figure 4). Considering that phosphorylation plays an important role in regulating TR3 activity, we generated the TR3(Q) mutant, in which, all of the serine residues, threonine residues and tyrosine residue within the fragment 41-61 are mutated to the non-phosphorylable residues. Our results indicate that phosphorylation of one or more of these residues are required for the function of TR3 in angiogenic responses (Figure 5). Paulsen *et al.* reported that mutation of serine 54 and threonine 55, partially, and mutation of both 54 and 55 amino acid residues greatly lose the transcriptional activity of TR3 in luciferase reporter assays [54]. However the functional consequence of these mutations in the angiogenic response of TR3 was not examined. Our data show that angiogenic responses are completely lose in the TR3(Q) mutant, suggesting that (a) our finding is consistent with the results from Paulsen *et al.* and (b) other phosphorylable amino acid resides in the 41-61 fragment are important for the function of TR3 in angiogenesis.

It was well known that TR3/Nur77 has several different functions which appear to depend on its cellular localization (reviewed in [31]). Previously, we reported that deletion of the transactivation domain (TR3ΔTAD) localizes in nuclei as TR3 full-length cDNA does [16], suggesting that all of the mutants in the transactivation, TR3Δ(1-20), TR3Δ(21-40), TR3Δ(41-60), TR3Δ(61-80), TR3Δ(101-120) and TR3(Q) should localize in nuclei. Although deletion of the transactivation domain (TR3ΔDBD) localizes in cytosol because the nuclear localization segment (NLS) of TR3 is in the DNA binding domain [16], our results clearly indicate that TR3(S351A), TR3(S351D) and Nur77ΔGRR are detected in nuclei (Figure 3F). Therefore, the inability of TR3 mutants to induce angiogenesis is not due to alteration of their cellular localization. Since we know that S351 is located within the DNA binding domain of TR3 and the fragment 41-60 is located within the transactivation domain, phosphorylation or dephophorylation of a site within one domain should not have any effect on the phosphorylation state of the other domain. Therefore, dephosphorylation of S351 and phosphorylation of Thr 48, 55 and 61, Ser 52 and 54, and Tyr 60 can happen simultaneously. Although several kinases regulate the phosphorylation of TR3 within the DNA binding domain and the transactivation domain, respectively, there is no known kinase/phosphatase that directly binds to TR3. We will identify the TR3 interacting proteins in future studies.

Unlike the DNA binding domain and the ligand-binding domain of TR3, the crystal structures of which were experimentally determined [18, 46], there is no reported structure of the N-terminal transactivation domain of TR3. These studies where we utilized computational approaches to evaluate the binding potential of residues within the N-terminal domain of TR3 and predict the structure of the transactivation domain, suggest sub-regions of binding potential within the N-terminal domain including the region 53-63 which correlates very well with our mutation studies within the overlapping 41-61 region. The Nur77, Nurr1, and NOR-1 (NR4A1-3 subgroup) family of orphan nuclear receptors is well conserved in the DNA binding domain (∼91-95%) and the C-terminal ligand binding domain (∼60%), but it is divergent in the N-terminal region that encodes the activation function-1 domain, AF-1. This serine/threonine-rich domain has been implicated in the regulation of transcription, as well as in the recruitment of cofactors including p300, SRCs, and PCAF and SHP-1 [55-57], among others (reviewed in [58]). The NR4A1-3 subgroup presents an exciting structure-function challenge where fully understanding the molecular mechanisms that mediate NR4A-dependent transcription will provide a potential platform for pharmacological intervention. However, S351 is in the DNA binding domain and the fragment 41-60 is in the transactivation domain. It is not possible to predict the orientation of the modeled N-terminal transactivation domain relative to the experimentally determined DNA-binding domain structure nor the interaction between these two domains by computational modeling, in the absence of protein crystallographic or nuclear magnetic resonance (NMR) data.

In summary, our previous studies demonstrated that TR3 is an excellent and specific target for therapies of pro-angiogenesis and anti-angiogenesis and that TR3 regulates angiogenesis in early stage by destabilization of the EC-EC interaction and modification of the EC-BM interaction via regulation of VE-cadherin associated adherence and integrin β4, respectively [16, 17, 29]. Current studies extend these results to demonstrate that TR3 regulates the formation of actin stress fibers, which is independent of RhoA. These studies further elucidate the structure and functional relationship of TR3 in angiogenesis by identification of novel amino acid residues that are critical for the function of TR3 in endothelial cells. Our current studies set the foundation for identification of compound(s) to inhibit or promote TR3 transcriptional activity for anti-angiogenic and pro-angiogenic therapies.

## MATERIALS AND METHODS

### Materials

VEGF was purchased from R&D Systems (Minneapolis, MN). Histamine, serotonin and antibody against Flag (Cat. No. F-3165) were purchased from Sigma (St. Louis, MO). Antibodies against TR3/Nur77 (Cat. No. sc-5569) and RhoA were obtained from Santa Cruz Biotechnology, Inc. (Santa Cruz, CA, USA). Rhodamine phalloidin was purchased from Invitrogen (Grand Island, NY). Endothelial cell basal medium (EBM), EGM-MV BulletKit, Trypsin/EDTA, and trypsin neutralization solution were obtained from Lonza (Allendale, NJ). Vitrogen 100 was purchased from Collagen Biomaterials (Palo Alto, CA, USA).

### Cell culture

Primary HUVECs purchased from Lonza (Allendale, NJ) were grown on plates coated with 30μg/ml vitrogen in endothelial basic medium (EBM) with EGM-MV BulletKit. HUVECs (passages 5) were used for all experiments.

### immunofluorescence staining and actin staining

HUVECs were seeded on collagen-coated glass coverslips in 24-well plates. Twenty-four hours later, cells were transduced with virus as indicated. Three days after infection, cells were serum starved for 24 hours, then rinsed with phosphate buffer saline (PBS), fixed with 4% paraformadehyde in PBS for 5 min, and incubated with 0.1 M glycine-PBS for 5 min. Cells were permeabilized with PBS containing 0.1% Triton X-100 for 2 min, incubated in blocking buffer (5% donkey serum-PBS) for 1 h, and then incubated with rhodamine phalloidin together with or without Alexa Fluor^®^ 488 Conjugated Deoxyribonuclease I (Life Technologies, Grand Island, NY) diluted in blocking buffer for 1 hour, rinsed with PBS, and mounted on glass slides with mounting medium containing DAPI (Vector) for photography by fluorescence or bright field microscopy.

### Proliferation assay

HUVECs (2 × 10^3^ cells/well) were seeded in 96-well plates. Twenty-four hours later, cells were transduced with viruses as indicated. Forty-eight hours later, cells were serum-starved with 0.1% FBS in EBM medium for 48 hours, cells were washed with PBS twice and kept in the −80°C freezer for several hours. A proliferation assay was carried out with CyQuant Cell Proliferation Assay Kit (Invitrogen, California) following the protocol provided by the producer. Data are expressed as the mean ± SD of quadruplicate values.

### Monolayer migration assay

The HUVEC monolayer migration assay was carried out as described [15]. Confluent HUVECs that were transduced with viruses as indicated on 6-well plates were subjected to serum starvation as above. The monolayer was then wounded with a single pass of a 200 μl pipette tip, washed with PBS, and photographed. Cells then were incubated for 16 hours, washed with PBS and photographed (Olympus BX 50). Data are expressed as number of cells migrating into the wounded area. Data are expressed as the mean ± SD of 6 views from three independent experiments.

### Monolayer permeability assay

The HUVEC monolayer permeability assay was carried out as described [17]. HUVECs (3.5 × 10^5^ per 24 wells) were seeded on 0.3 μm pore size polycarbonate membranes in HTS transwell-24 well plates (Corning Incorporated, Corning, NY). Twenty-four hours later, cells were transduced with viruses as indicated. Two days later, culture medium was replaced. Twenty-four hours later, fluoresceinated-dextran, 70 KDa (Molecular Probes, Eugene, Oregon), was added to the transwell at a final concentration of 0.25 mg/ml. FITC-dextran in the bottom wells was read after 1 hour with SoftMax Pro program (excitation: 494 nm, emission: 518 nm, cutoff: 515 nm) in a Spectra Max M5 instrument (Molecular Devices Corporation, Sunnyvale, CA).

### Computational protein structure modeling and binding site potential analysis

The sequence from human TR3, residues 1-167, was computationally assessed for protein secondary structure prediction with PSIPRED Protein Sequence Analysis [47], protein binding region prediction in disordered proteins (ANCHOR [48-50]), interface residue predictions (BSpred [51]) and protein structure prediction (GalaxyWEB [52]). The results from all predictions were correlated and rendered on representations of the predicted structure of the N-terminal transactivation domain using YASARA [59].

### Statistical analysis

Results are presented as mean ± SD. Student's t-test was employed to determine statistical significance. P values less than 0.05 were considered to be statistically significant.

### Construction of mutants

#### N-terminal deletion mutants

TR3Δ(1-20) cDNA was generated by PCR with pTOPO-TR3 plasmid as template [16], the forward primer TR3Δ(1-20)-61F and the reverse primer TR3ΔLBD-1101R. The PCR product was digested with XhoI and XmnI and cloned into the corresponding site of pTOPO-TR3 to form pTOPO-TR3Δ(1-20). The TR3Δ(1-20) was then subcloned to pMF vector [16] with XhoI and BamHI restriction enzymes.

TR3Δ(21-40) cDNA was created by two rounds of PCR. In the first round of PCR, two DNA fragments, A) TR3(1-60bp, Δ21-40) and B) TR3(120-1392bp,Δ21-40) were generated using plasmid pTOPO-TR3 as the template and primer pairs A) TR3-1F and TR3Δ(21-40)-60R, and B) TR3Δ(21-40)-120F and TR3-1392R, respectively. In the TR3-1F primer, an XhoI enzyme restriction sequence was inserted just before the translation starting site (ATG). In the reverse primer TR3Δ(21-40)-60R, the nucleotide sequence ending at amino acid 20 was fused to a 10-base sequence of TR3 beginning at amino acid 41. In TR3Δ(21-40)-120F oligonucleotide, a 10-base sequence of TR3 ending at amino acid 20 (underlined) was fused with amino acid 41 of TR3. TR3-1392R oligonucleotide contains a BamHI restriction site. In the second round of PCR, the PCR products of A) TR3(1-60bp, Δ21-40) and B) TR3(120-1392bp, Δ21-40) were used as templates and oligonucleotides TR3-1F and TR3-1392R as primers. The second-round PCR product was digested with restriction enzymes XhoI and NgoMIV and replaced the corresponding fragment in pMF-TR3 to generate pMF-TR3Δ(21-40). TR3Δ(41-60), TR3Δ(61-80) and TR3Δ(101-120) were created in the similar way as TR3Δ(21-40) with primers listed in Supplemental Table 1S. All of the annealing temperatures for these PCRs are 60°C.

#### pMF-TR3(S351A) and pMF-TR3(S351D)

Plasmid pMF-TR3(S351A) was generated by one run of PCR as outlined in Supplemental Figure 7S. TR3(1034-1797bp, S351A) DNA fragment was generated using pMF-TR3 as the template and oligonucleotides TR3(S351A)-1034F and TR3-1797R as primers. The PCR product TR3(1034-1797bp, S351A) was digested with restriction enzyme NgoMIV and BamHI and replaced the corresponding wild type fragment in pMF-TR3 to generated pMF-TR3(S351A). pMF-TR3(S351D) were generated in the same ways with primer TR3(S351D)-1034F.

#### Generation of pMF-Nur77(S142A), pMF-Nur77(S142D), pMF-Nur77(S144A), pMF-Nur77(S144D), pMF-Nur77(T145A), pMF-Nur77(T145E) and Nur77ΔGRR mutants

Because there are none suitable restriction enzyme recognition sites found in TR3 to be used to generate the mutants, we generated the mutants of serine 142, serine 144 and threonine 145 in mouse Nur77 cDNA. Plasmid pMF-Nur77(S142A) was generated by two runs of PCR as outlined in Supplemental Figure 8S. In the first-round PCR, two DNA fragments, Nur77(292-435bp,S142A) and Nur77(415-1059bp, S142A), were generated using pMF-Nur77 as the template and oligonucleotides Nur77-292F and Nur77(S142A)-435R; Nur77(S142A)-435F and Nur77-1059R as primers respectively. These two PCR products were used as templates in the second-round PCR, using Nur77-292F and Nur77-1059R as primers to generate Nur77(292-1059bp,S142A) fragment. The PCR product Nur77(292-1059bp,S142A) was digested with SmaI/NgoMIV and replaced the corresponding wild type fragment in pMF-Nur77 to generated pMF-Nur77(S142A). Because there are two NgoMIV restriction digestion sites in Nur77 cDNA, the pMF-Nur77 was digested with restriction enzymes 1) SmaI and NheI, and 2) NgoMIV and NheI to obtained two fragments for sub-cloning. pMF-Nur77(S142D), pMF-Nur77(S144A), pMF-Nur77(S144D), pMF-Nur77(T145A), pMF-Nur77(T145E) were generated in the same ways with primers, Nur77(S142D)-435F and Nur77(S142D)-435R for pMF-Nur77(S142D), Nur77(S144A)-435F and Nur77(S144A)-435R for pMF-Nur77(S144A), Nur77(S144D)-435F and Nur77(S144D)-435R for pMF-Nur77(S144D), Nur77(T145A)-435F and Nur77(T145A)-435R for pMF-Nur77(T145A), Nur77(T145E)-435F and Nur77(T145E)-435R for pMF-Nur77(T145E), respectively. Nur77ΔGRR mutant was generated by the non-specific priming of Nur77-1059R during the PCR construction of the Nur77(S142A) mutant.

#### Generation of point mutant TR3(Q)

Point mutant TR3(Q), that contains 6 mutated amino acids, T48A-S52A-S54A-T55A-Y60F-T61A, was created by 2 steps as outlined in Supplemental Figure 9S. In the first step, two plasmids, pMF-TR3(T48A) and pMF-TR3(Y60F-T61A) were generated (Supplemental Figure 9S, A). Plasmid pMF-TR3(T48A) was generated by two runs of PCR. In the first-round PCR, two DNA fragments, TR3(1-152bp,T48A) and TR3(132-1392bp,T48A), were generated using pTOPO-TR3 as the template and oligonucleotides TR3-1F and TR3(T48A)-152R; TR3(T48A)-132F and TR3-1392R as primers respectively. These two PCR products were used as templates in the second-round PCR, using TR3-1F and TR3-1392R as primers to generate TR3(1-1392bp,T48A) fragment. PCR product TR3(1-1392bp, Y60F-T61A) fragment was generated in the similar manner, using TR3-1F and TR3(Y60F-T61A)-191R; TR3(Y60F-T61A)-171F and TR3-1392R, respectively, as the first-round PCR primers to generate PCR fragments TR3(1-191bp, Y60F-T61A) and TR3(171-1392bp, Y60F-T61A). In the 2^nd^ PCR, the 1^st^ PCR products, TR3(1-191bp, Y60F-T61A) and TR3(171-1392bp, Y60F-T61A), were used as template and oligonucleotides TR3-1F and TR3-1392R as primers. During the second-round PCR, the first three cycles employed 52°C as annealing temperature while others employed 60°C. The PCR product TR3(1-1392bp,T48A) and TR3(1-1392bp,Y60F-T61A) were digested with XhoI/NgoMIV and replaced the corresponding wild type fragment in pMF-TR3 to generated pMF-TR3(T48A) and pMF-TR3(Y60F-T61A), respectively. In the 2^nd^ step, the N-terminal portion, TR3(1-175bp,T48A-S52A-S54A-T55A) was generated by PCR, using pMF-TR3(T48A) as the template, oligonucleotides TR3-1F and TR3(52A-54A-55A)-175R as primers (Supplemental Figure 9S, B). The C-terminal portion, TR3(148-1392bp,S52A-S54A-T55A-Y60F-T61A) was amplified, using PMF-TR3(Y60F-T61A) as the template, TR3(52A-54A-55A)-148F and TR3-1392R as primers. The TR3(1-175bp,T48A-S52A-S54A-T55A) and the TR3(148-1392bp,S52A-S54A-T55A-Y60F-T61A) were used as templates in the subsequent PCR, using oligonucleotides TR3-1F and TR3-1392R as primers. The PCR product was termed as TR3(1-1392bp,T48A-S52A-S54A-T55A-Y60F-T61A) that was digested with restriction enzymes XhoI and NgoMIV to replace the corresponding wild type fragment in pMF-TR3 to generate plasmid pMF-TR3(Q) containing T48A-S52A-S54A-T55A-Y60F-T61A mutant.

All of the mutants were confirmed by DNA sequencing and are expressed as Flag or Flag-HA in-frame fused proteins. All the sequences of primers are listed in Supplemental Table 2S.

### Construction and preparation of adenovirus vectors expressing TR3 and its mutants

Plasmid pMIG purchased from Addgene (Cambridge, MA) was digested with restriction enzyme XhoI, blunted with DNA polymerase I large Klenow fragment, and then digested with restriction enzyme SalI. The 1.3 Kb DNA fragment containing the IRES-hGFP from pMIG was cloned to pENTR1A obtained from Invitrogen (Grand Island, NY), which were digested with restriction enzyme EcoRI, blunted with DNA polymerase I large Klenow fragment and then digested with restriction enzyme XhoI, to obtain plasmid pENTR1A-MIG. pENTR1A was digested with restriction enzymes XmnI and NotI. pENTR1A-MIG was digested with restriction enzymes BamHI and NotI to obtain the 1.3 Kb IRES-GFP fragment. FH-DSCR1-1L DNA fragment was obtained from pMFH-DSCR1-1L [60] that was digested with restriction enzymes PmlI and BamHI, because TR3 cDNA contains PmlI restriction site. These three DNA fragments were ligated to obtained plasmid, pENTR1A-MIG-FH-DSCR1-1L, which contains the Flag-HA in-frame fused with DSCR1-1L in the N-terminus and the GFP protein is expressed by internal ribosome entry site (IRES). The DSCR1-1L cDNA was replaced with TR3 cDNA digested from pMF-TR3 with restriction enzymes XhoI and BamHI. All of the TR3 mutants were subcloned to pENTR1A-PMIG vector in the same way. These pENTR1A-PMIG vectors were used with the pAd/CMV/V5-DEST™ Gateway^®^ Vector (Cat. No. V493-20) obtained from Invitrogen (Grand Island, NY) to construct and prepare adenovirus following the instruction provided by the company.

## SUPPLEMENTARY MATERIAL TABLES AND FIGURES


