# Supplementary material for: Requirement of novel amino acid fragments of orphan nuclear receptor TR3/Nur77 for its functions in angiogenesis
Source: Oncotarget. 2015 Jun 25;6(27):24261–76. doi: 10.18632/oncotarget.4637 (PMC4695184; doi:10.18632/oncotarget.4637)
Supplement: Supplementary file 1 [file oncotarget-06-24261-s001.pdf]

Requirement of novel amino acid fragments of orphan nuclear receptor TR3/Nur77 for its functions in angiogenesis

Supplementary Material

**Table 1S. Generation of N-terminal deletion mutants of TR3**				
**Mutants**	**1st PCR primers**	**1st PCR product**	**2nd PCR primers**	**2nd PCR product**
TR3∆(21-40)	**A)** TR3-1F and TR3∆(21-40)-60R	**A)** TR3(1-60bp, ∆21-40)	TR3-1F and TR3-1392R	TR3(1-1392bp, ∆21-40)
	**B)** TR3∆(21-40)-120F and TR3-1392R	**B)** TR3(120-1392bp, ∆21-40)		
TR3∆(41-60)	**A)** TR3-1F and TR3∆(41-60)-120R	**A)** TR3(1-120bp, ∆41-60)	TR3-1F and TR3-1392R	TR3(1-1392bp, ∆41-60)
	**B)** TR3∆(41-60)-180F and TR3-1392R	**B)** TR3(180-1392bp, ∆41-60)		
TR3∆(61-80)	**A)** TR3-1F and TR3∆(61-80)-180R	**A)** TR3(1-180bp, ∆61-80)	TR3-1F and TR3-1392R	TR3(1-1392bp, ∆61-80)
	**B)** TR3∆(61-80)-240F and TR3-1392R	**B)** TR3(240-1392bp, ∆61-80)		
TR3∆(101-120)	**A)** TR3-1F and TR3∆(101-120)-300R	**A)** TR3(1-300bp, ∆101-120)	TR3-1F and TR3-1392R	TR3(1-1392bp, ∆101-120)
	**B)** TR3∆(101-120)-360F and TR3-1392R	**B)** TR3(360-1392bp, ∆101-120)		

Table 2S. Primer sequences

Primer	Sequence
TR3∆(1-20)-61F	GCTCGAGCTGGCAAGCGACCCCCTGACC
TR3∆LBD-1101R	CGGATCCTTAATCCAGGTGTGCAAGGACCAGG
TR3-1F	GGTTGCCTCGAGATGCCCTGTATCCAAGCCCAATAT
TR3-1392R	CGGATCCTTAGAAGATGAGCTTGCCCTCGCCTG
TR3∆(21-40)-60R	GGGCTGCCTCGTGGTCACGGGGTCCCGGAC
TR3∆(41-60)-120R	ACTCTCCTGTGGGGCTGGCCAGGTCCATGG
TR3∆(61-80)-180R	AGGAGGAGGCGTAGCCGTCCATGAAGGTGC
TR3∆(101-120)-300R	GGGCCTCATCGGAGGCAGAGGCAGGGGAGG
TR3∆(21-40)-120F	CCGTGACCACGAGGCAGCCCCCGCTGCCCC
TR3∆(41-60)-180F	GGCCAGCCCCACAGGAGAGTTTGACACCTT
TR3∆(61-80)-240F	GGACGGCTACGCCTCCTCCTCGGCCTCCTC
TR3∆(101-120)-360F	CTCTGCCTCCGATGAGGCCCTGTCCTCCAG
TR3(T48A)-132F	CGCTGCCCCCGCTGCCCTGCC
TR3(T48A)-152R	GGCAGGGCAGCGGGGGCAGCG
TR3(52A-54A-55A)-148F	CTGCCCGCCTTCGCCGCCTTCATGGACG
TR3(52A-54A-55A)-175R	CGTCCATGAAGGCGGCGAAGGCGGGCAG
TR3(60F-T61A)-171F	GGACGGCTTCGCAGGAGAGTT
TR3(60F-T61A)-191R	AACTCTCCTGCGAAGCCGTCC
Nur77(S142A)-435F	CCCCGCGCCATCTACACCCAACTTCCAG
Nur77(S142A)-435R	TGGAAGTTGGGTGTAGATGGCGCGGGGG
Nur77(S142D)-435F	CCCCGACCCATCTACACCCAACTTCCAG
Nur77(S142D)-435R	TGGAAGTTGGGTGTAGATGGGTCGGGGG
Nur77(S144A)-435F	CCCCTCGCCAGCTACACCCAACTTCCAG
Nur77(S144A)-435R	TGGAAGTTGGGTGTAGCTGGCGAGGGGG
Nur77(S144D)-435F	CCCCTCGCCAGACACACCCAACTTCCAG
Nur77(S144D)-435R	TGGAAGTTGGGTGTGTCTGGCGAGGGGG

Nur77(T145A)-435F	CCCCTCGCCATCTGCACCCAACTTCCAG
Nur77(T145A)-435R	TGGAAGTTGGGTGCAGATGGCGAGGGGG
Nur77(T145E)-435F	CCCCTCGCCATCTGAACCCAACTTCCAG
Nur77(T145E)-435R	TGGAAGTTGGGTTCAGATGGCGAGGGGG
Nur77-1059R	TGGGTTTTGAAGGTAGCCGGCCCCGCCG
Nur77-292F	CCCGCTTCGGCGGACTTCAAGTTTGAGG
TR3(S351A)-1034F	GGCGGGGCCGGCTACCTGCAAAACCCAAGC
TR3(S351D)-1034F	GGCGGGGCCGGCTACCTGACAAACCCAAGC
TR3-1797R	CCCATCGGATCCTCAGAAGGGCAGCGTGTCCATGAA

Figure 1S. Diagram of TR3 domains and the deletion mutants

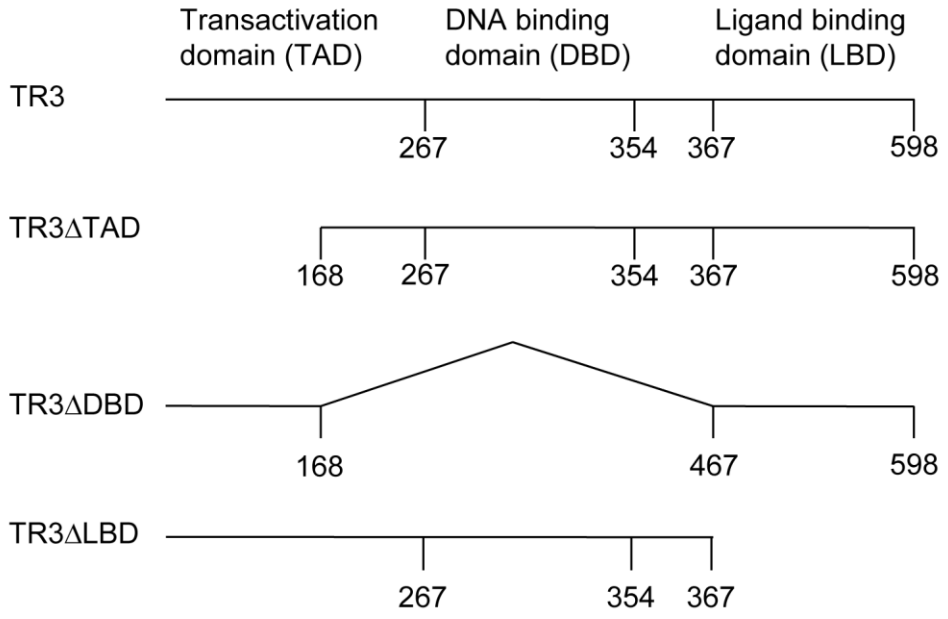

Figure 2S. Photographs of migration assay for Figure 2C

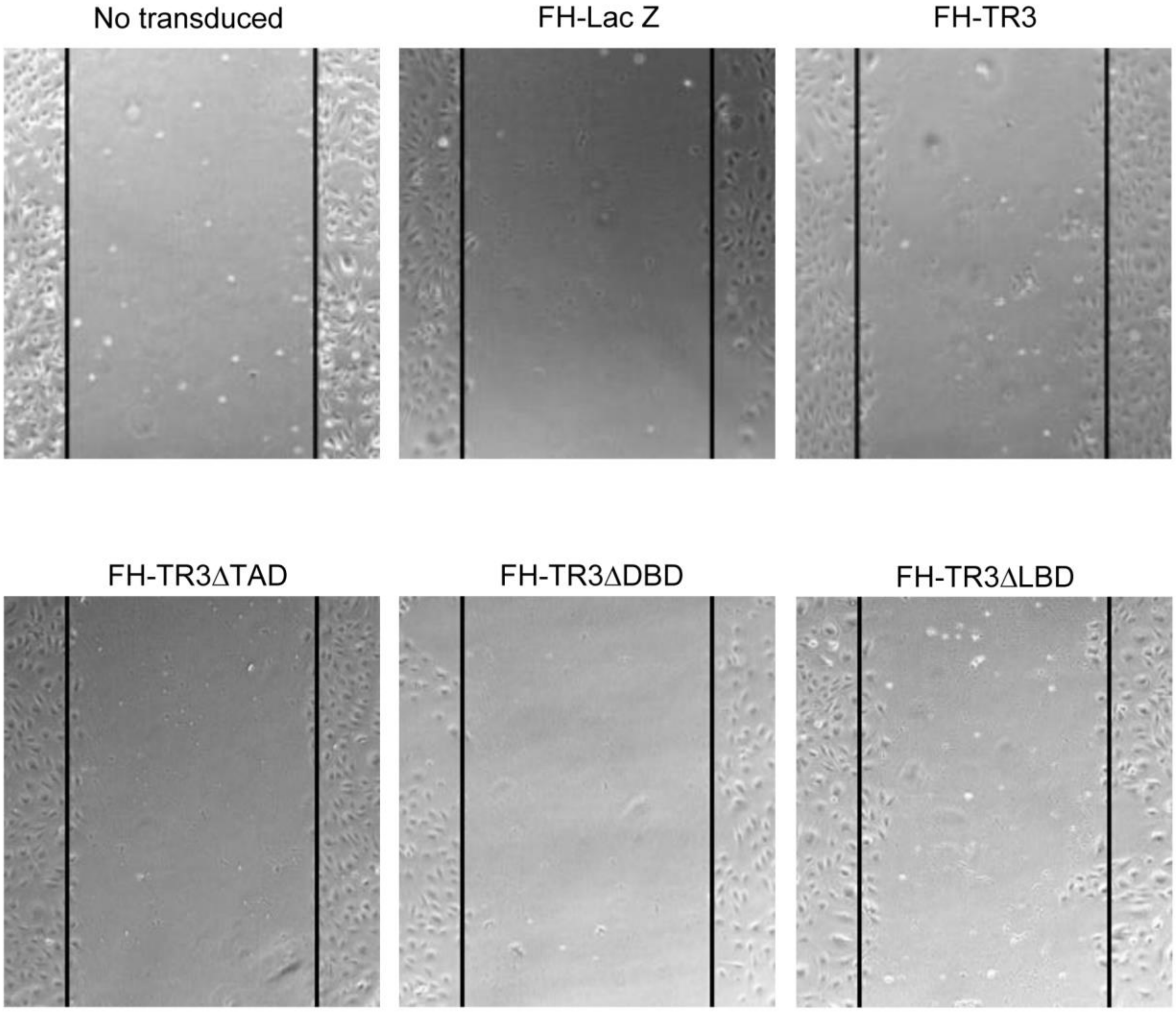

Figure 3S. Morphology HUVEC transduced with mutants as indicated

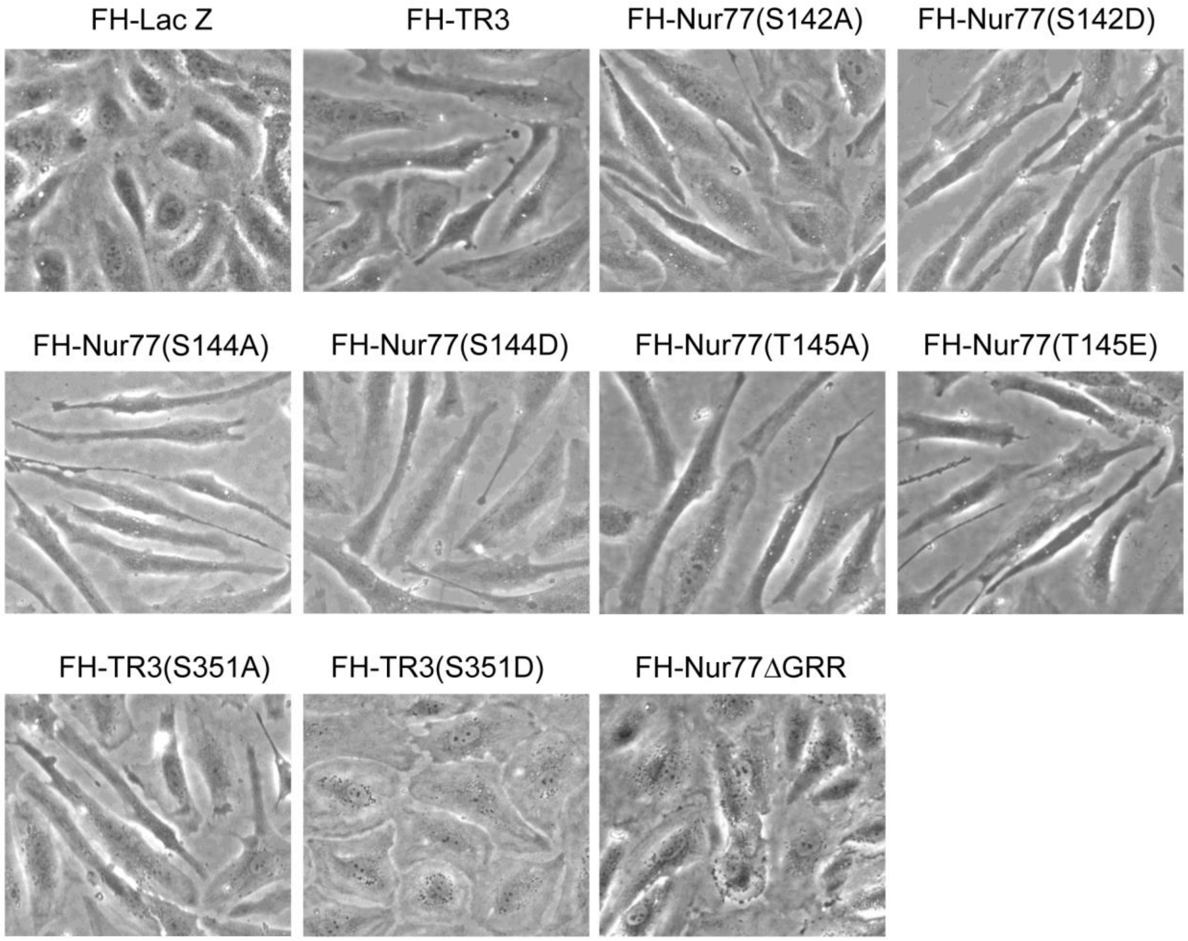

Figure 4S. Photographs of migration assay for Figure 3D

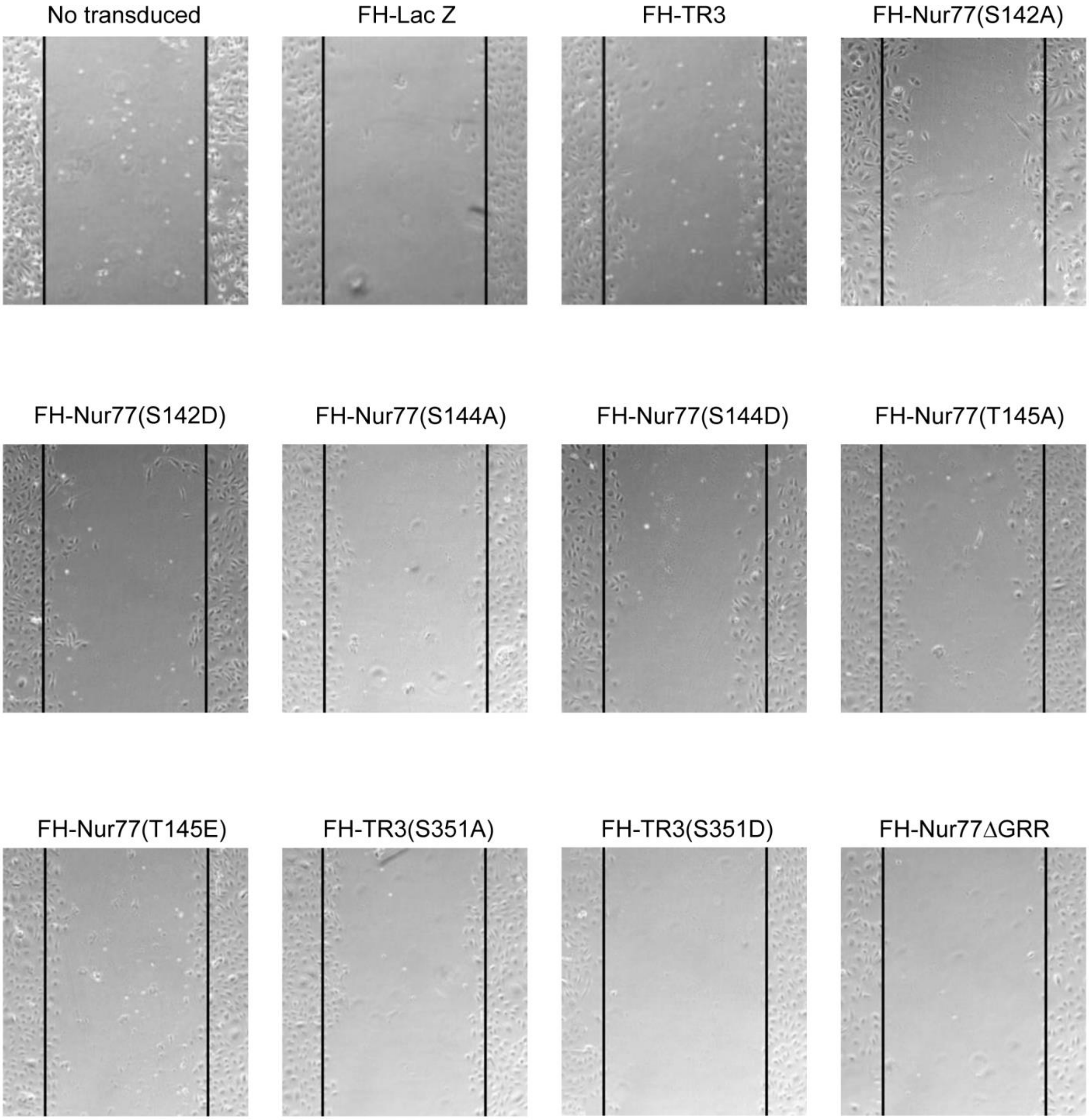

Figure 5S. Photographs of migration assay for Figure 4E

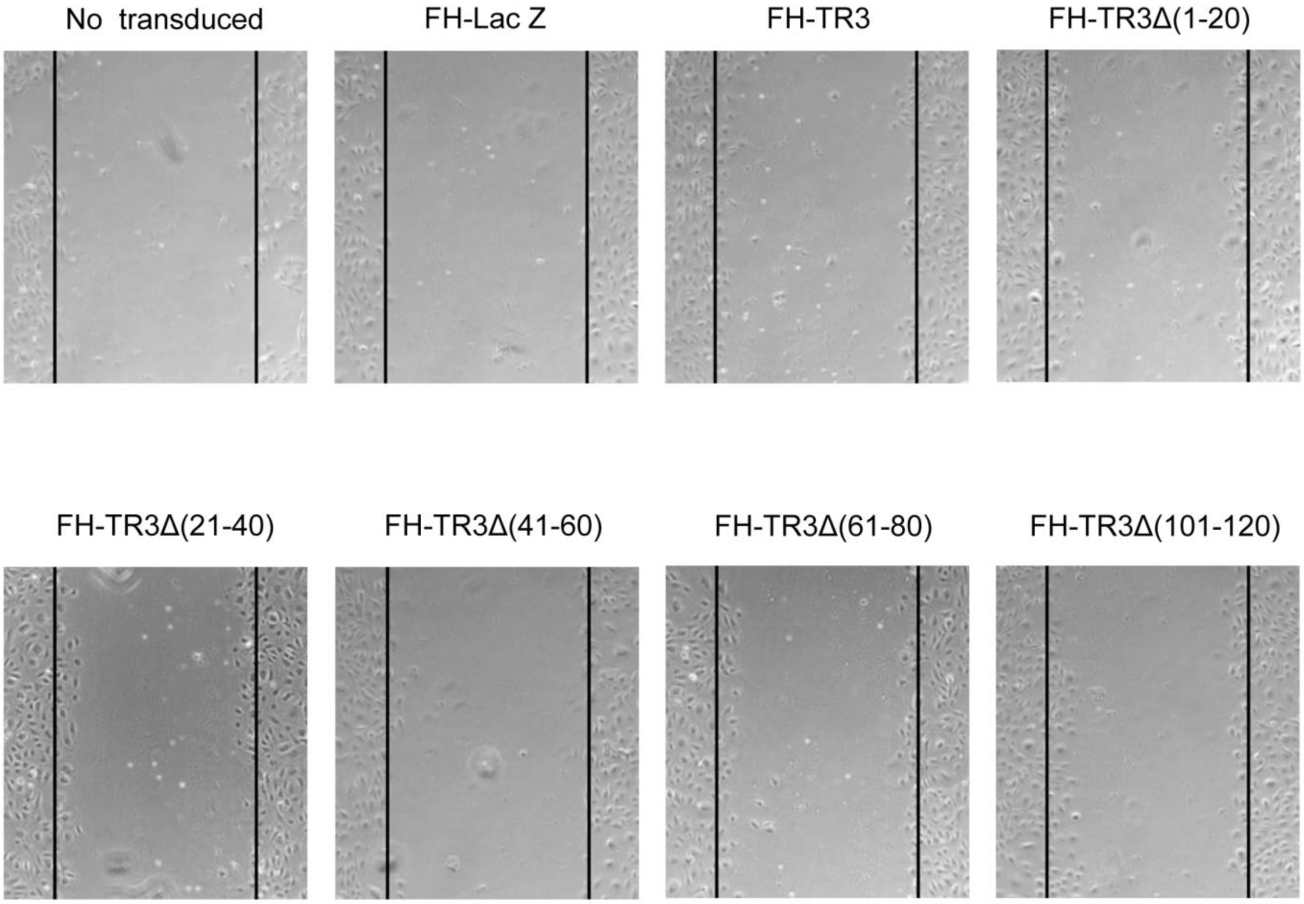

Figure 6S. Photographs of migration assay for Figure 5D

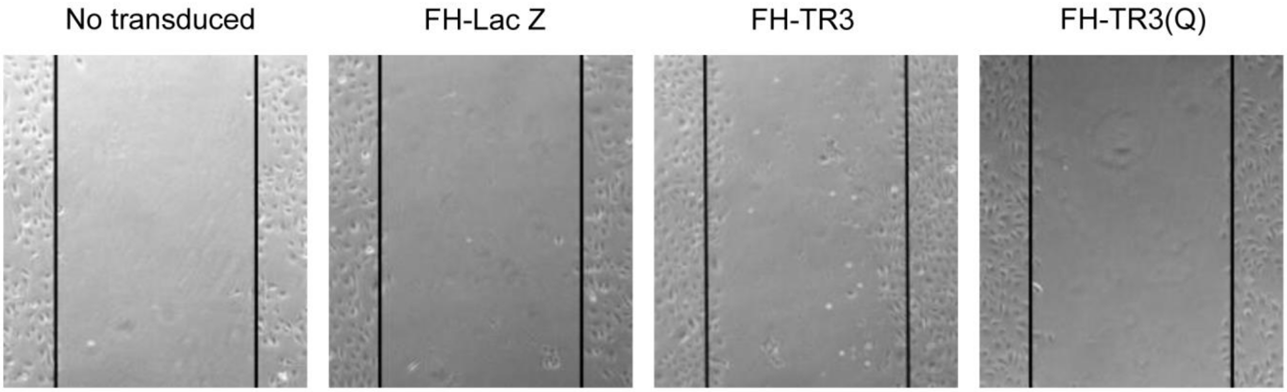

Figure 7S. Diagram of construction of pMF-TR3(S351A)

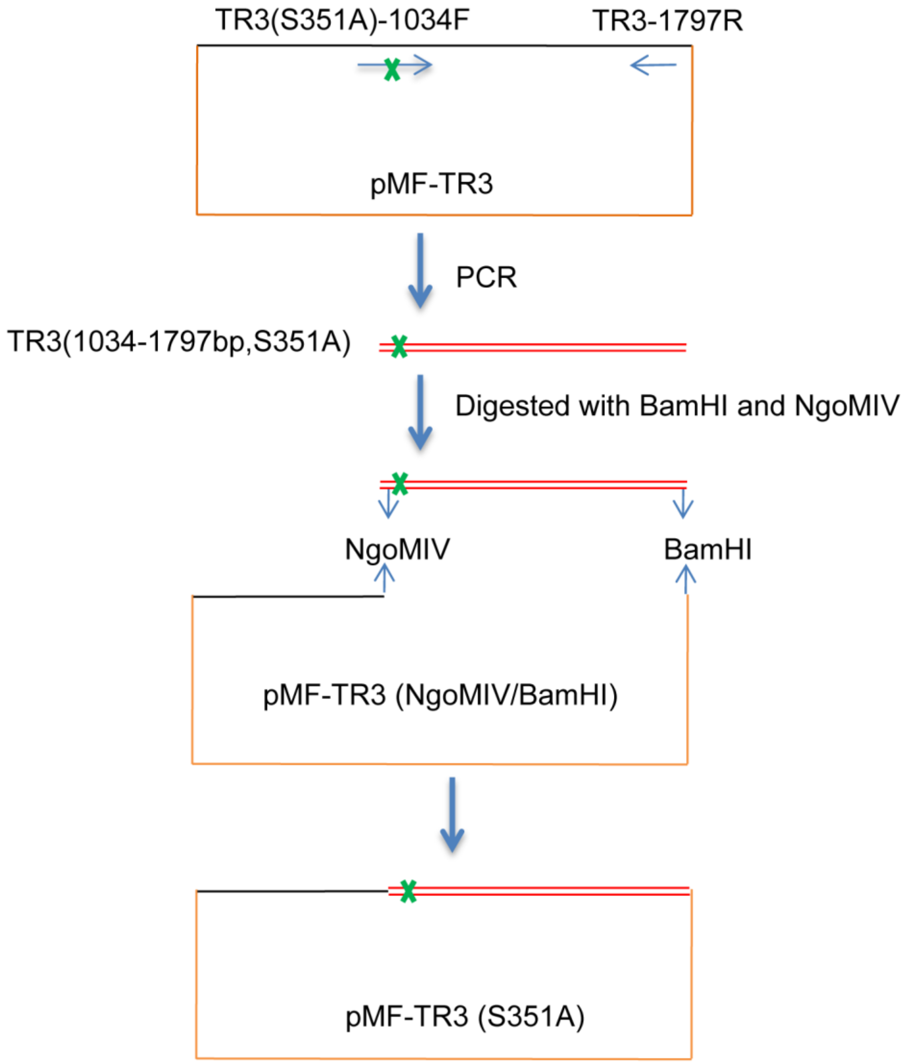

Figure 8S. Diagram of construction of pMF-Nur77(S142A)

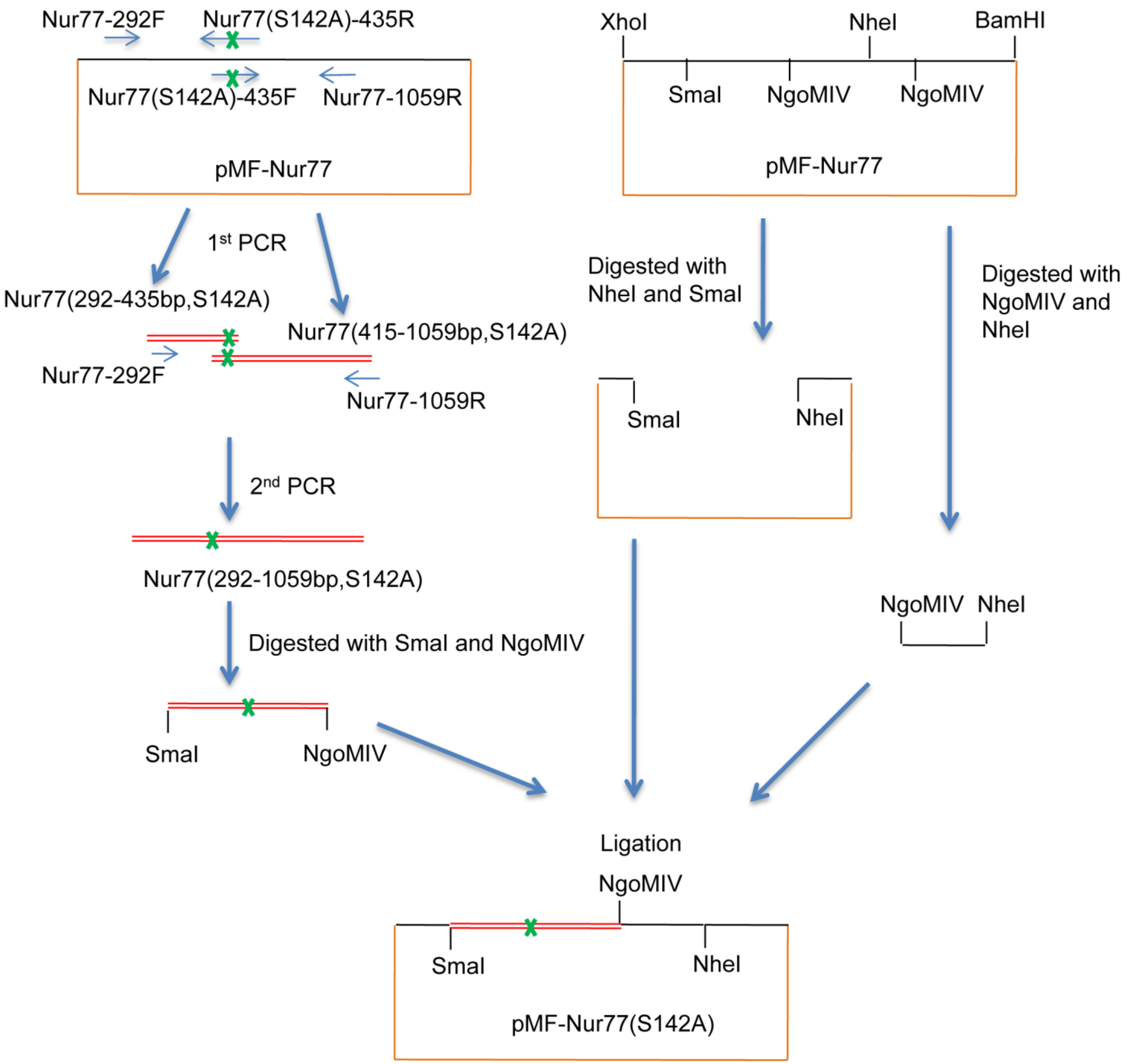

Figure 9S. Diagram of construction of pMF-TR3(Q)

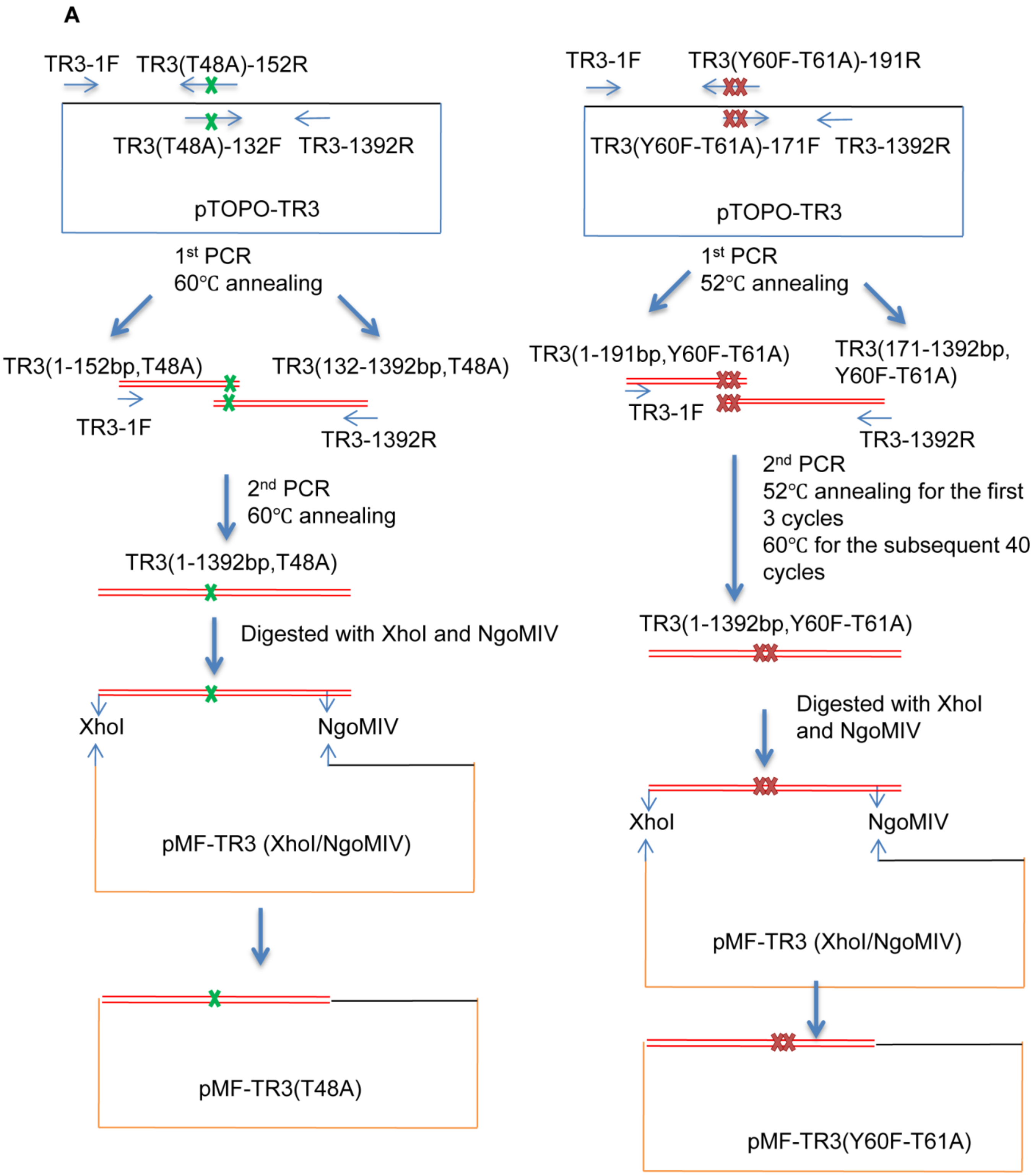

B

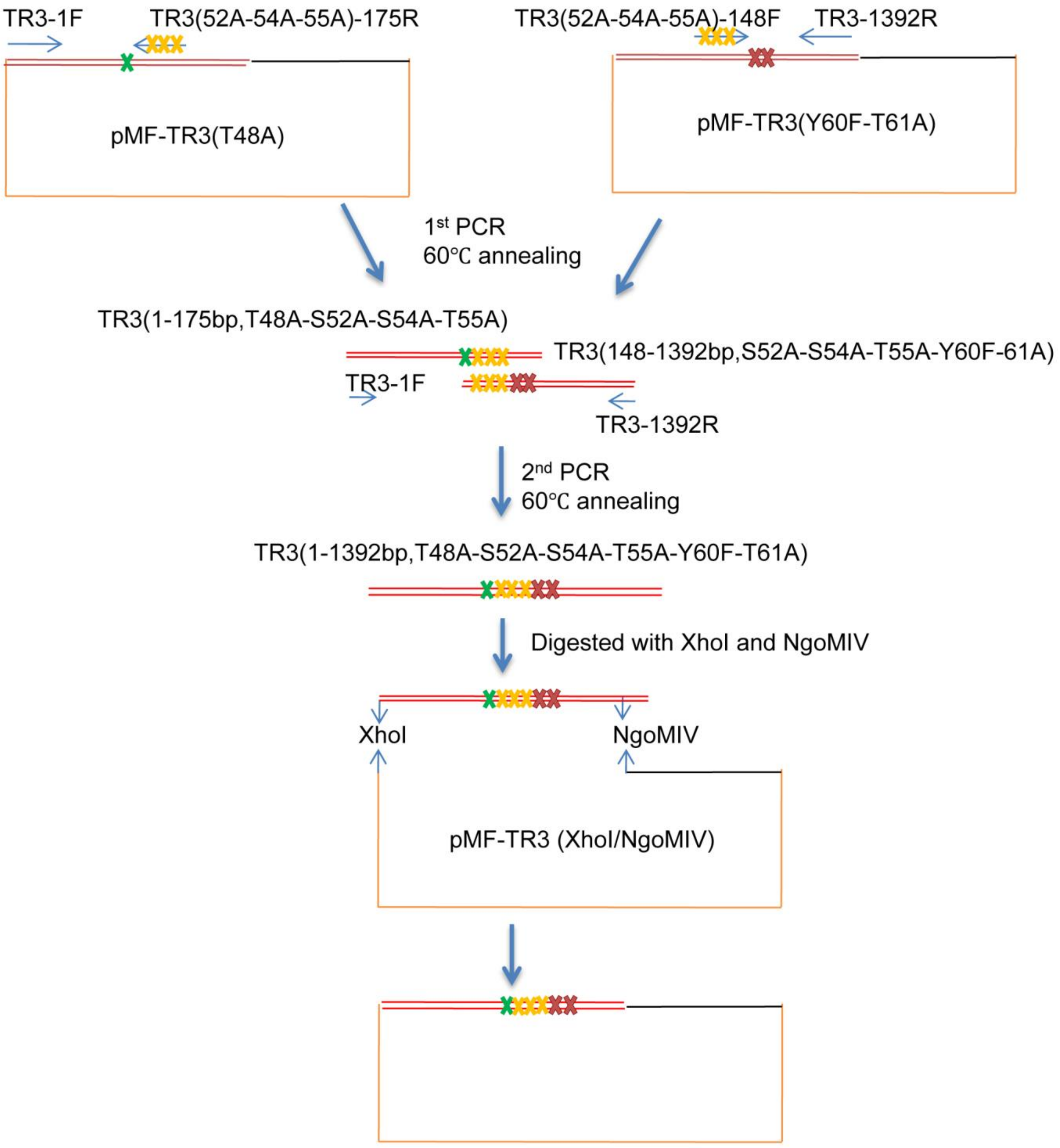

pMF-TR3(Q) is pMF-TR3(T48A-S52A-S54A-T55A-Y60F-T61A)
